# The Ætiology of Rickets

**Published:** 1908-10-31

**Authors:** 


### Diseases of Children.

### THE ÆTIOLOGY OF RICKETS.

Year after year the medical profession devotes itself to the reduction of its sources of income by ascertaining the causes of disease and the means of prevention. Rickets is very much a case in point, for it is a grave source of alimentary and respiratory affections in infancy, of nervous phenomena less directly, and of serious deformities which may affect the individual throughout life, and in the future mother prove a source of disaster during confinement.

Yet although much has been done in the prevention of rickets as the result of better methods of hygiene, suitable dietary, and exercise, it is still a problem in ætiology as to what is the main factor in the production of this widely spread disease, which has probably existed since the world began, though it was not described until the seventeenth century by Glisson.

There is little evidence of heredity, though Hausen states that a stallion begat seven rachitic foals and that two of the mares subsequently had healthy foals by other sires. Antenatal factors are of importance. The child may be delicate and more prone to the disease on account of deterioration of the mother's health during pregnancy. Deficient lactation consequent on too frequent pregnancies, insufficient diet, and worry or other causes may influence normal pregnancy unfavourably. It is noteworthy that there is not often evidence of the disease before the sixth month of life, a fact suggestive of post-natal causes, though not excluding the possibility of antenatal factors predisposing to or even initiating a slowly developing affection such as this.

Geographical and sociological considerations help us a little. Rickets is most common in the temperate zone; rare in the tropics, China, Japan, and the southern parts of Italy and Spain; almost unknown near the equator, in Iceland, Greenland, Norway, Denmark, and Greece; rare at high altitudes. It is much more common in towns than in country districts. Negroes and Italians in New York are very prone to the disease, although in their own countries it is rare. This statement must be modified in so far as the affection is becoming more and more common in Italian towns. It is almost as common among the upper as the lower classes, though in a less severe form. All this evidence suggests that maternal nursing and open-air life in a mild climate have more influence in the prevention of the disease than mere locality. Confinement in ill-ventilated houses is thought an important factor. The worst cases in England are generally seen in the spring months, following the confined life and damp climate of winter. But it must be pointed out that in some more northern countries, in which the winter is longer and the child kept even more indoors, the disease is rare or non-existent. Perhaps this is partly due to breast feeding or to a more liberal diet of fatty foods.

Findlay has recently brought forward evidence that lack of exercise is a potent factor. Experimental animals and birds kept in cages often develop rickets, although they get plenty of sun and fresh air. He was unable to induce rickets in puppies by diet if they were allowed free exercise, whereas he developed it in others under suitable diet if they were merely kept in confinement. Further he pointed out that it is difficult, if not impossible, to cause rickets by diet without confinement. This theory is supported by the rarity of the disease in hot countries, and in Japan, in spite of prolonged lactation, its greater frequency in winter months, and its prevalence in town rather than country. On the other hand Guérin fed puppies on a meat diet and they became markedly rachitic, while others of the same litter, suckled, showed no sign of the disease. Bland Sutton found that young lions weaned early and fed on raw meat only became very rachitic, but they were cured by adding milk and cod liver oil to the diet. The animals were allowed plenty of exercise. Two young monkeys fed on a purely vegetarian diet also became rachitic.

It is important to be certain that the affection produced in animals by diet is true rickets and not some other osseous change, analogous to osteomalacia, the result of deficiency of lime salts or other constituent of the food. It is equally important to bear in mind that many feeding experiments fail to produce rickets in young animals although the animals die from the profound marasmus set up. Marasmic infants do not become rachitic, or, at any rate, they do not exhibit the osseous and cartilaginous changes characteristic of the disease. Apparently marasmus and rickets are more or less incompatible. To test the truth of Findlay's theory that the disease is due to lack of exercise it is necessary that the diet of the animals should be such as not to set up a state of marasmus. The best marked cases in infants are those of very fat babies who have been fed on condensed milks or proprietary foods, rich in sugar and deficient in fat and proteid. Possibly the few cases seen in the breast-fed are due a maternal milk supply with similar defects, usually a deficiency in fat.

Thus, even after many years of study, we can only ascribe the disease to dietetic errors, combined with bad hygienic surroundings and lack of fresh air and exercise. The latter factors may act by limiting the proper metabolism of the food taken. I suggest, therefore, that an excess of carbohydrate food is a potent cause, and that deficient metabolism leads to the production of by-products which seriously affect the cartilages and bones, as well as the general metabolism of the body. If this be so, the disease is the result of a luxus consumption of a particular food or a relative excess in proportion to the other constituents of the diet. We see diseased states set up in a similar way in cases of diabetes and conditions of acidosis. Here the restriction in carbohydrate food, often combined with an excess of fatty food, leads to disordered metabolism and the occurrence of acetonuria which will frequently disappear on the addition of starchy food to the diet.

It is possible that further chemical researches will reveal indications of erroneous metabolism, when the carbohydrates largely exceed the fat in the diet.

Though digestive disorders are often present in the rachitic they are a sequel rather than a cause, for there is no reliable evidence that the disease is due to microbial infection or intestinal toxæmia. Intestinal disorders set up marasmus rather than rickets. I doubt whether either syphilis or tuberculosis have any influence beyond reducing the resisting power of the child. More research is clearly necessary before we thoroughly understand the pathogeny of this disease, but the practical outcome of our present knowledge is that babies must be fed on a diet containing suitable proportions of *all* the different food constituents, that they should have plenty of air and sunlight, and be allowed free movement of their limbs.

